# Do Acupuncture Services Reduce Subsequent Utilization of Opioids and Surgical Interventions Compared to Noninvasive Therapies among Patients with Pain Conditions?

**DOI:** 10.1093/pm/pnab187

**Published:** 2021-06-15

**Authors:** Timothy Pham, Qinli Ma, Abiy Agiro, Julie Bukowiec, Terry Flannery

**Affiliations:** 1 HealthCore, Inc., Wilmington, Delaware; 2 Office of Medical Policy and Technology Assessment, Anthem Inc., Latham, New York, USA

**Keywords:** Acupuncture, NSAIDs, Opioids, Pain Medicine, Physical Therapy, Surgical Procedures

## Abstract

**Objective:**

To compare prescribed opioid use and invasive surgical interventions between patients using acupuncture and those using non-steroidal anti-inflammatory drugs (NSAIDs)/physical therapy (PT).

**Design:**

Retrospective observational study of administrative claims.

**Setting:**

Large commercial insurance plan.

**Subjects:**

52 346 each treated with either acupuncture or NSAIDs/PT.

**Methods:**

Users of acupuncture and NSAIDs/PT were identified from January 1, 2014, to December 31, 2017. The first date of each service was defined as the index date. Acupuncture patients were 1:1 propensity score matched to the NSAIDs/PT group on baseline characteristics. Outcomes included opioid use, subsequent invasive surgical procedures, healthcare utilization such as hospitalizations or emergency department (ED) visits, and costs. These were assessed in the 12-month period before index date (baseline) and 12-month period following index date (follow-up) using difference-in-difference (DID) analysis. Results for opioid use were stratified by those with and without baseline opioid use.

**Results:**

The acupuncture group had fewer patients initiating opioids post-index both among those with (49.2% vs 56.5%, *P* < .001) and without (15.9% vs 22.6%, *P* < .001) baseline opioid use. There was a small increase in invasive surgical procedures with acupuncture (3.1% vs 2.8%, *P* = .006). A reduction in ED visits was observed with acupuncture (DID −4.6% for all-cause; −3.3% for pain-related, all *P* < .001). Acupuncture was associated with higher total medical and pharmacy costs (DID +$1331 per patient, *P* = .006).

**Conclusions:**

Acupuncture showed a modest effect in reducing opioid use and ED visits. More research on acupuncture’s place in emergency care, pain relief, and comparison to other types of non-opioid treatment is needed.

## Introduction

Data from national health surveys indicate that 50 million American adults (20.4% of the population) suffer from chronic pain [1]. In response to the high burden of chronic pain, prescriptions for opioids increased by 300% from 1999 to 2012 [2, 3]. Exposure to prescription opioids is linked to risk of developing an opioid addiction [4] and subsequent morbidity and mortality [5].

Due to deaths and disease burden from the opioid epidemic, US public health organizations and policy makers recommend increased use of nonopioid and nonpharmacologic therapies for chronic pain. Recommended alternatives include cognitive behavioral therapy, physical therapy (PT), nonsteroidal anti-inflammatory drugs (NSAIDs), and acupuncture [6, 7]. Some evidence shows that acupuncture may be an effective alternative for treating chronic pain [8]. Proponents suggest that it may decrease reliance on opioids, and thus may reduce opioid addiction [9]. However, many health plans do not provide a benefit for acupuncture services, many on the basis of an inadequate evidence base [10]. Interestingly, the Centers for Medicare and Medicaid services (CMS) announced in January 2020 that it would cover acupuncture for chronic low back pain in Medicare beneficiaries, citing the potential for acupuncture to reduce overuse of and addiction to opioids [11]. Their rationale includes it as an option among a range of therapies that rely less on prescription opioids [11].

Acupuncture, a procedure involving manual or electric (electroacupuncture) stimulation of specific points on the body using sterile needles, has long been used in East Asia for various conditions [12]. In the United States, around 3.5 million adults receive acupuncture treatment annually [12]. Chronic neck or back pain and headache are the most common conditions for which it is prescribed [13]. Studies using animal models have shown acupuncture to have analgesic effects by stimulating the release of endogenous opioids, but the therapeutic mechanisms remain unclear [14, 15].

Although acupuncture has been proposed to reduce exposure to opioids, there are few studies that have examined its effect on lowering prescribed opioid use. A meta-analysis of 13 articles found that acupuncture reduced opioid analgesic usage in the postoperative setting [16]. One study by Zheng et al. (2008) compared opioid consumption by 35 patients with chronic pain who received electroacupuncture and others who received sham acupuncture. This study found no statistically significant difference between the two groups [17]. A follow-up randomized controlled trial by Zheng et al. (2019) compared electroacupuncture to sham electroacupuncture or education alone for 108 patients with chronic musculoskeletal pain. This study found no difference in the reduction of opioid dosage among the three groups [18]. A study using the Vermont Medicaid population, reported that a majority of patients with chronic pain who received acupuncture treatments self-reported reduced use of opioid medications [19]. These studies are constrained by their relatively small populations, limited geographic regions, and use of self-reported measures. Other studies have examined the effects of acupuncture on symptoms of opioid use disorder, but not opioid utilization [20, 21]. To build upon previous studies, using a more nationally representative population, we describe here prescribed opioid use and the use of invasive surgical interventions for members of a large commercial insurance plan using acupuncture compared to a matched group of patients using physical therapy and/or NSAIDs without acupuncture. We also compared healthcare utilization and healthcare costs between the two groups.

## Methods

We used administrative medical and pharmacy claims data from the HealthCore Integrated Research Environment (HIRE^SM^) to capture clinical, utilization, and health plan/patient paid cost measures (as opposed to charged amounts or projected cost). The HIRE is a repository of fully adjudicated claims data for commercially insured and Medicare Advantage insured members from 14 health plans with membership across the entire United States. This observational study, conducted under the Research Exception provisions of Privacy Rule 45 CFR 164.514(e), was exempt from Institutional Board Review because researchers accessed a limited dataset for analysis which was devoid of individual patient identifiers, and complied with all relevant provisions of the Health Insurance Portability and Accountability Act. Institutional review board exemption was not necessary because the study was an analysis of the managed care organization’s membership data for the purposes of health plan treatment, planning, and operations.

This retrospective cohort study included patients 18 years of age or older who had at least one claim with a *Current Procedural Terminology (CPT)* or *Generic Product Identifier (GPI)* code for acupuncture or other therapies (i.e., nonsteroidal anti-inflammatory drugs [NSAIDs], physical therapy [PT]) between January 1, 2014, and December 31, 2017 (Supplementary Appendix Table 1 for list of codes). NSAIDs and/or physical therapy were chosen as comparison therapies because they are commonly used non-opioid treatments for chronic pain. The first date of service for acupuncture use or NSAIDs/PT use was set as the index date. At least 12 months of continuous medical and pharmacy coverage before and after the index date was required. Additionally, at least one diagnosis of neck pain, back pain, headache, or migraine within 90 days prior to index was required. These diagnoses were chosen because they are the most common pain conditions for which acupuncture is used in the data. Patients with any cancer diagnosis in the 12 months prior to index were excluded, since opioid use is likely to be different than the general population in terms of dosing and length of treatment. Acupuncture users could not have had any acupuncture service within 1 year prior to the index date, regardless of whether they used NSAIDs or PT. Patients using NSAIDs and/or PT could not have had claims for NSAIDs/PT within 1 year prior to the index date, nor could they have had any acupuncture service during the study period. A subgroup analysis compared acupuncture users to physical therapy users alone as both treatments involve a licensed practitioner applying physical manipulation.

Outcome measures were assessed in both the 12-month pre-index period (baseline) and 12-month post-index period (follow-up). The primary outcomes consisted of opioid use and follow-up incidence of any invasive surgical procedures for neck/back pain or headache/migraine. Measured parameters of opioid use included any opioid use, number of fills, dose in morphine milligram equivalents (MME) per day, and thresholds for 50 MMEs/day. Invasive surgical procedures (Supplementary Appendix 1) identified through CPT codes were only examined in the follow-up period. Secondary outcomes consisted of utilization measures that included interventional pain procedures, all-cause and pain-related hospitalizations, ED visits, physician office visits, and imaging tests. All-cause and pain-related costs for healthcare utilization were also assessed. Claims with any pain condition in any of the diagnosis fields were considered pain-related utilization except for hospitalizations and ED visits, which required a pain condition in the primary diagnosis field.

Baseline demographics included age, sex, region, insurance plan type, urban/rural residence, as well as additional information obtained at 9-digit zip code level (race/ethnicity, median household income, and level of education). Baseline clinical characteristics consisted of the Deyo-Charlson Comorbidity Index Score [22], mental health disorders (depression, anxiety, substance use disorders, other mental health), pain diagnoses (back, neck, headache/migraine, joint, fibromyalgia/myositis, pelvic, extremity, temporomandibular disorder, neuropathic pain, fractures, contusions, injuries, kidney stones/gallstones, chest, rheumatoid arthritis, osteoarthritis, sickle cell), other nonsurgical interventional pain procedures (chiropractor, osteopathic manipulation treatment, transcutaneous electrical stimulation [TENS], neurolytics), and number of unique medications (sum of GPI-8 codes).

To reduce confounding, we used a propensity score matching (PSM) method to make comparison groups more similar. A logistic regression model generated propensity scores using baseline age, sex, region, payor type, urban residence, index year, race/ethnicity, median income, education level, Deyo-Charlson Comorbidity score, back pain, joint pain, fibromyalgia, pelvic pain, extremity pain, injuries, chiropractor use, transcutaneous electrical nerve stimulation (TENS) use, opioid use, inpatient visits, ED visits, number of medications, and medical costs. The greedy nearest neighbor matching technique (an algorithm that chooses a treatment group member and then chooses a control group member that is the closest match) was used to create a 1:1 matched cohort [23]. Before and after PSM, balance in baseline characteristics between comparison groups was assessed with standardized differences (d). Absolute standardized differences greater than 0.10 were considered statistically significant.

Outcomes were assessed using the difference-in-difference (DID) framework within matched groups. Differences from baseline to follow-up within the acupuncture group were compared to differences from baseline to follow-up within the NSAIDs/PT group. For the opioid measures, results were stratified by baseline opioid use in order to clearly delineate those who did and did not initiate opioids after index acupuncture or other therapy. Regression using generalized estimating equations compared outcomes with log-link and binomial distribution for dichotomous outcomes, negative binomial distribution for count outcomes, and gamma distribution for cost outcomes. Absolute changes in DID format were reported, along with adjusted *P* values for cohort and time interaction. *P* values less than .05 were considered statistically significant. All analyses were conducted using SAS Enterprise Guide version 7.1 (SAS Institute, Cary, NC).

## Results

**Figure 1.**
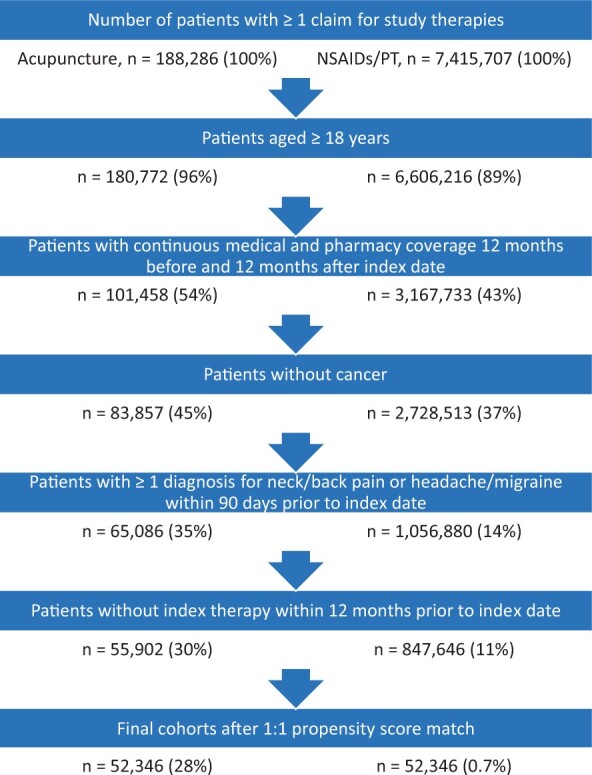
Flow chart of patient attrition. NSAIDs = nonsteroidal anti-inflammatory drugs; PT = physical therapy.

A total of 55,801 patients treated with acupuncture and 845,656 patients treated with NSAIDs/PT were identified after applying inclusion and exclusion criteria (see Figure 1**)**. Unmatched, the acupuncture and NSAIDs/PT groups differed significantly (d > 0.1) in many demographic characteristics. The acupuncture cohort were more likely to be female (65.1% vs 53.3%, d = 0.20), live in the Western census region (63.0% vs 22.8%, d = 0.89), reside in an urban residence (90.0% vs. 74.1%, d = 0.42), be Asian (13.3% vs 4.7%, d = 0.61), have a higher median household income ($108,264 vs $83,430, d = 0.52), and have a higher number of pain diagnoses (2.4 vs 1.9, d = 0.25). Of the main pain diagnoses of interest, neck pain was the most different (26.2% vs 19.1%, d = 0.17). Baseline opioid use was similar (24.2% vs 25.1%, d = 0.02) (Supplementary Appendix Table 2 for further descriptive statistics of the unmatched groups). After 1:1 matching, the two groups had similar baseline characteristics, with 52,346 patients in each group. For example, standardized differences in age, sex, Charlson comorbidity score, neck pain, back pain, headache/migraine and baseline opioid use were <0.1 (see Table 1 for more details).

**Table 1.** Baseline demographics and clinical characteristics of patients using acupuncture and nonsteroidal anti-inflammatory drugs/physical therapy (NSAIDs/PT) after propensity score matching

	Acupuncture	NSAIDs/PT	Standardized Difference
Sample size, n	52,346	52,346	
Age, years, mean (SD)	45.6 (13.17)	45.5 (15.64)	<0.01
Female, n (%)	33,825 (64.6)	33,667 (64.3)	<0.01
Geographic region, n (%)			0.06
Northeast	13,488 (25.8)	13,696 (26.2)	
West	32,760 (62.6)	32,815 (62.7)	
Midwest	1,610 (3.1)	1,451 (2.8)	
South	3,199 (6.1)	3,008 (5.7)	
Missing/Unknown	<10	<10	
Insurance plan type, n (%)			0.14
CDHP	7,530 (14.4)	7,706 (14.7)	
HMO	5,368 (10.3)	5,392 (10.3)	
PPO	39,447 (75.4)	39,248 (75.0)	
Other	<10	<10	
Urban/rural classification based on zip code, n (%)			<0.01
Urban	47,298 (90.4)	47,230 (90.2)	
Rural	3,791 (7.2)	3,779 (7.2)	
Missing/Unknown	1,257 (2.4)	1,337 (2.6)	
Proportions of Race/Ethnicity based on zip code, mean % (SD)			
Asian	10% (17%)	10% (17%)	<0.01
Black	10% (12%)	10% (12%)	<0.01
White	70% (24%)	70% (24%)	<0.01
Hispanic	20% (22%)	20% (22%)	<0.01
Household income based on zip code, median (IQR)	$107,481 ($53,481)	$105,471 ($51,102)	0.03
Deyo-Charlson Comorbidity Index Score, mean (SD)	0.3 (0.83)	0.3 (0.80)	<0.01
Deyo-Charlson Comorbidity Index Score Categories, n (%)			0.06
0	40,606 (77.6)	40,234 (76.9)	
1	8,157 (15.6)	8,488 (16.2)	
2	2,208 (4.2)	2,262 (4.3)	
3+	1,375 (2.6)	1,362 (2.6)	
Comorbidities of interest, n (%)			
Depression	6,513 (12.4)	6,531 (12.5)	<0.01
Anxiety	8,411 (16.1)	7,861 (15.0)	0.03
Substance use disorders	699 (1.3)	716 (1.4)	<0.01
Other mental health	6,058 (11.6)	5,513 (10.5)	0.03
Number of pain diagnoses, mean (SD)	2.3 (2.11)	2.3 (1.95)	<0.01
Relevant pain diagnoses			
Back pain	24,253 (46.3)	24,117 (46.1)	<0.01
Neck pain	12,917 (24.7)	12,781 (24.4)	<0.01
Headache/migraine	8,305 (15.9)	9,509 (18.2)	<0.01
Any opioid use, n (%)	12,446 (23.8)	12,205 (23.3)	0.01
Hospital admissions, mean (SD)	0.1 (0.4)	0.1 (0.3)	<0.01
ED visits, mean (SD)	0.2 (0.7)	0.2 (0.7)	<0.01
Number of unique medications (GPI-8), mean (SD)	5.3 (5.3)	5.3 (5.1)	0.01

CDHP = consumer-driven health plan; ED = emergency department; GPI-8 = Generic Product Indicator-8 digits; HMO = health maintenance organization; PPO = preferred provider organization; SD = standard deviation.

**Figure 2.**
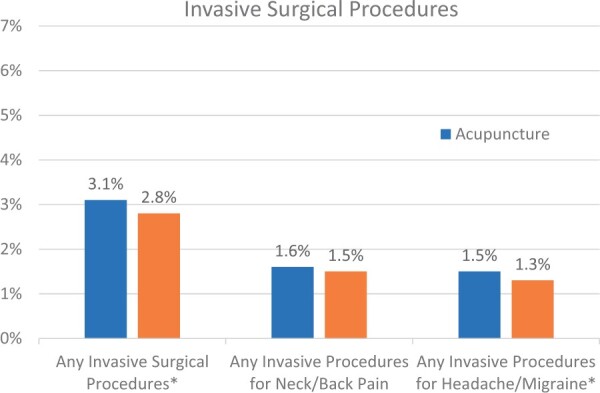
Effect of acupuncture on post-index invasive surgical therapies. *Statistically significant different at .05 level.

Table 2 shows the effect of acupuncture on measures of opioid use, stratified by baseline opioid use, from baseline to follow-up period with the matched groups. Those without baseline opioid use (naive opioid users) numbered 39,900 patients in the acupuncture group, of which 15.9% initiated opioids post-index, and 40,141 patients in the NSAIDs/PT group, of which 22.6% initiated opioid post-index (*P* < .001). Both number of fills (1.6 fills vs. 1.7 fills, *P* < .001) and total days of supply (12.9 days vs. 14.5 days, *P* = .004) for opioids were lower for the naïve opioid acupuncture group. However, total MME/day (37.8 MME/day vs 36.7 MME/day, *P* < .001) and the number reaching the greater than 50 MME/day threshold (24.0% vs 22.0%) were higher. For those with baseline opioid use, there were 12,446 patients in the acupuncture group, of which 49.2% continued use post-index, while of the 12,205 patients in the NSAIDs/PT group, 56.5% continued use post-index (*P* < .001). Among these continuous opioid users, number of opioid fills remained stable among the acupuncture group (+0.0 fills) while increasing for the NSAIDS/PT group (+0.4 fills, DID −0.4, *P* < .001). Otherwise no statistically significant differences were seen with the other opioid measures. Patients using acupuncture showed slightly higher use of post-index invasive surgeries (Figure 2, 3.1% vs 2.8%, *P* = .006).

**Table 2.** Effect of acupuncture on opioid use and dosage stratified by baseline opioid use

	Acupuncture			NSAIDs/PT			DID AbsoluteDifference	
	BaselinePeriod	Follow-upPeriod	AbsoluteDifference	BaselinePeriod	Follow-upPeriod	AbsoluteDifference		
Naive opioid users (without baseline opioid use)								
	Acupuncture (n = 39,900)			NSAIDs/PT (n = 40,141)				*P* value*
Number of members with opioid use post-index, n (%)	6,333 (15.9%)			9,068 (22.6%)			−6.7	<.001
Number of fills, mean (SD)	…	1.6 (1.4)	…	…	1.7 (1.6)	…	−0.1	<.001
Total days' supplied, mean (SD)	…	12.9 (25.7)	…	…	14.5 (33.0)	…	−1.6	.004
Total quantity dispensed, mean (SD)	…	62.7 (119.8)	…	…	66.4 (139.8)	…	−3.7	.450
Total MME per day, mean (SD)	…	37.8 (23.6)	…	…	36.7 (23.5)	…	1.1	<.001
Above 50 MME per day threshold, n (%)	…	1518 (24.0%)	…	…	1996 (22.0%)	…	2.0	<.001
Continuous opioid users (with baseline opioid use)								
	Acupuncture (n = 12,446)			NSAIDs/PT (n = 12,205)				Adjusted *P* value^†^
Number of members with opioid use post-index, n (%)	6,127 (49.2%)			6,897 (56.5%)			−7.3	<.001
Number of fills, mean (SD)	5.1 (5.7)	5.1 (5.6)	0.0	5.4 (6.4)	5.8 (6.4)	0.4	−0.4	<.001
Total days' supplied, mean (SD)	108.1 (173.0)	113.1 (176.9)	5.0	125.1 (198.1)	133.1 (201.6)	8.0	−3.0	.296
Total quantity dispensed, mean (SD)	409.5 (693.4)	418.7 (680.3)	9.2	496.0 (923.0)	516.3 (898.2)	20.3	−11.1	.276
Total MME per day, mean (SD)	44.9 (77.3)	48.4 (185.3)	3.5	53.9 (114.2)	53.3 (107.8)	−0.6	4.1	.092
Above 50 MME per day threshold, n (%)	1,431 (23.4%)	1,595 (26.0%)	2.6%	1,835 (26.6%)	1,935 (28.1%)	1.5%	1.1	.084

a *P* values generated from Wilcoxon-Mann-Whitney tests for continuous measures and χ^2^ goodness of fit tests for categorical measures.

† Adjusted *P* values for interaction term of therapeutic group and time were outputted from generalized estimating equation models.

DID = difference in difference; MME = morphine milligram equivalents; NSAIDs = nonsteroidal anti-inflammatory drugs; PT = physical therapy; SD = standard deviation.

Healthcare utilization measures are presented in Table 3. The acupuncture group was associated with a small increase in all-cause hospitalizations (+2.7% vs +2.1%, DID +0.7%, *P* = 0.01), but not in pain-related hospitalizations (+0.8% vs. 1.0%, DID −0.2%, *P* = .54). All-cause and pain-related ED visits decreased in the acupuncture group relative to NSAIDs/PT (−1.7% vs +2.9%, DID −4.6% for all-cause and −1.3% vs +2.0%, DID −3.3% in pain-related, respectively, *P* < .001 for both). Additionally, acupuncture use was associated with lower use of non-surgical interventional pain procedures (DID −0.7%, *P* < .001). Patients in the acupuncture group had increased medical and pharmacy costs relative to those in the PT/NSAIDs group (+$3,725 vs +$2,394, DID +$1,331 per patient, *P* = .006). These were driven by increases in total (all-cause) medical costs (+$3,269 vs +$2,230, DID +$1,039, *P* = 0.01). However, acupuncture was also associated with a decrease in average ED costs (−$78 vs +$93, DID −$171, *P* < .001).

**Table 3.** Effect of acupuncture on healthcare utilization and total costs

	Acupuncture (n = 52,346)				NSAIDs/PT (n = 52,346)			DID Absolute Difference	Adjusted *P* value*
	Baseline Period	Follow-up Period	Absolute Difference		Baseline Period	Follow-up Period	Absolute Difference		
All-cause								
Inpatient hospitalization								
N (%)	3,323 (6.3)	4,756 (9.1)	2.7%	3,257 (6.2)	4,331 (8.3)	2.1%	0.7%	.0
Count, mean (SD)	0.1 (0.4)	0.1 (0.5)	0.03	0.1 (0.4)	0.1 (0.5)	0.03	0.01	.19
ED visits								
N (%)	7,732 (14.8)	6,818 (13.0)	−1.7%	7,656 (14.6)	9,164 (17.5)	2.9%	−4.6%	<.001
Count, mean (SD)	0.2 (0.7)	0.2 (0.7)	−0.02	0.21 (0.7)	0.25 (0.8)	0.05	−0.07	<.001
Physician office visits								
N (%)	48,899 (93.4)	50,889 (97.2)	3.8%	50,243 (96.0)	50,675 (96.8)	0.8%	3.0%	<.001
Count, mean (SD)	7.4 (7.6)	9.8 (9.6)	2.4	6.6 (6.6)	7.8 (7.4)	1.2	1.2	<.001
Pain-related								
Inpatient hospitalization								
N (%)	1,426 (2.7)	1,855 (3.5)	0.8%	1,513 (2.9)	2,023 (3.9)	1.0%	−0.2%	.54
Count, mean (SD)	0.01 (0.1)	0.03 (0.2)	0.01	0.01 (0.2)	0.03 (0.2)	0.01	0.00	.67
ED visits								
N (%)	4,126 (7.9)	3,450 (6.6)	−1.3%	4,368 (8.3)	5,402 (10.3)	2.0%	−3.3%	<.001
Count, mean (SD)	0.1 (0.5)	0.1 (0.4)	−0.02	0.1 (0.4)	0.1 (0.5)	0.03	−0.04	<.001
Physician office visits								
N (%)	34,703 (66.3)	44,127 (84.3)	18.0%	36,547 (69.8)	42,421 (81.0)	11.2%	6.8%	<.001
Count, mean (SD)	2.8 (4.4)	4.7 (6.6)	1.9	2.2 (3.3)	3.1 (4.0)	0.9	1.0	<.001
Total all-cause costs^†^ per patient, mean (SD)								
Total medical and pharmacy costs	$9,884	$13,609	$3,725	$9,322	$11,716	$2,394	$1,331	.006
Medical costs	$8,686	$11,955	$3,269	$7,487	$9,717	$2,230	$1,039	.01
Hospitalization cost	$2,474	$3,415	$941	$2,480	$2,950	$470	$471	.02
ED cost	$594	$516	−$78	$571	$664	$93	−$171	<.001
Pharmacy costs	$1,904	$2,182	$278	$2,011	$2,280	$269	$9	.66

* Adjusted *P* values for interaction term of therapeutic group and time were outputted from generalized estimating equation models.

† Members with third-party pharmacy coverage excluded.

DID = difference in difference; ED = emergency department NSAIDs = nonsteroidal anti-inflammatory drugs; PT = physical therapy; SD = standard deviation.

The analysis of acupuncture users compared to PT only users included 51,428 patients in each group after PSM. No statistically significant differences were seen in naive opioid users initiating opioid use post-index (15.9% of acupuncture vs 16.0% of PT only, *P* = .551. However, number of opioid fills (1.59 fills vs 1.62 fills, *P* = .047), total quantity dispensed (61.8 units vs 65.7 units, *P* = .016), total MME/day (37.9 MME/day vs 39.2 MME/day, *P* = .003), and proportion reaching greater than 50 MME/day threshold (24.1% vs 25.9%, *P* = .016) were lower among the naive opioid acupuncture group. For those with baseline opioid use, no statistically significant differences were found among those initiating opioid use (48.9% of acupuncture vs 50.1% of PT only, *P* = .064). Supplementary Appendix Table 3 for more details. All-cause and pain-related ED visits decreased in the acupuncture group relative to PT (−1.6% vs –0.4%, DID -1.2% for all-cause, *P* < .001 and −1.2% vs. –0.7%, DID −0.5% in pain-related, *P* = .011). The acupuncture group showed relatively higher total medical and pharmacy costs (+$3,657 vs +$1,979, DID +$1,678, *P* < .001), mostly driven by medical costs (+$3,219 vs +$1,841, DID +$1,378, *P* < .001). Differences among ED visit costs were not statistically significant. Supplementary Appendix Table 4. Other results were similar to the main analysis.

## Discussion

This large retrospective cohort study compared clinically important utilization outcomes between acupuncture and other common non-opioid therapies for common pain conditions (neck/back pain or headache/migraine). Acupuncture users initiated opioids in smaller proportions than those who used NSAID/PT, even when stratified by baseline opioid use. No meaningful difference was seen on subsequent invasive surgical procedures. However, acupuncture showed a statistically and clinically significant reduction in all-cause and pain-related ED use in the follow-up period.

Our results indicate that acupuncture does appear to reduce opioid initiation for neck/back pain or headache/migraine compared to NSAIDs or physical therapy. Fewer members initiated opioids after starting acupuncture therapy, regardless of whether they were taking opioids beforehand. However, acupuncture members in the opioid-naive group who later used opioids tended to have slightly higher doses (∼1 MME/day) than the comparison group. No difference in dose change was found with members continuously using opioids. The number of fills and total days of supply were lower for the acupuncture group. These differences were statistically, but not clinically, significant. Overall, acupuncture showed an advantage in ceasing opioid use entirely rather than reducing use. These results differ from previous studies conducted by Zheng et al. that found little or no difference in opioid use with acupuncture [17, 18]. Our results may have varied from theirs due to their use of sham acupuncture or education as comparison groups, while we compared acupuncture to commonly used therapies in a real-world setting. We also used a larger sample of members who received acupuncture and had access to claims data, especially dose, on filled prescriptions for opioids.

In alignment with our findings, the Vermont Medicaid acupuncture study [19] found that 32% of opioid users reported a decrease in their opioid use following acupuncture treatment. As that study did not include a control group and only included self-reported outcomes, our results add additional information to their findings. Additionally, we include a longer 12-month follow-up period, in contrast to the Vermont study’s follow-up time of 60 days.

One notable finding was that the acupuncture group had a slight decrease in all-cause and pain-related ED visits, while the NSAIDs/PT group showed an increase in ED utilization. In the subgroup comparison between acupuncture and PT alone, both groups showed a reduction in ED use, but the average reduction in the acupuncture group was greater. To our knowledge, no study has examined acupuncture’s effect on use of emergency care. The mechanism for this benefit is unknown and further prospective research is needed to confirm this finding. In contrast to ED visits, there was a statistically significant increase in overall inpatient hospitalizations among acupuncture users compared to NSAIDs/PT, although this was small (<1%). Acupuncture users also showed slightly higher use of subsequent invasive surgical procedures. It is unknown whether these results were due to adverse effects from acupuncture treatment or from unmeasured confounding in baseline clinical characteristics.

Insurers play an important role in providing access to health care, but usually lack information on the clinical effectiveness of the services they cover [10] and coverage of multidisciplinary nonpharmacological pain therapies remain low in the United States [24]. Our study addresses a gap in knowledge regarding the benefits of acupuncture. We found evidence that acupuncture services reduced opioid use compared to NSAIDs/PT. For patients who were not using opioids before, fewer initiated opioid use after acupuncture. For patients who were using opioids before, more dropped opioid usage entirely after acupuncture. However, when comparing acupuncture to PT alone, these differences did not hold. Acupuncture also showed benefit in decreasing ED utilization but not inpatient hospitalization. Costs were generally higher for acupuncture and were driven by overall medical and hospitalization costs. The mean (SD) cost for acupuncture services over the follow-up period was slightly higher at $1,308 ($4,910) than for physical therapy at $1,294 ($10,928). Other considerations, such as adherence and clinical effectiveness, will play an important role in the long-term comparative costs of these services. The present study may inform employer decisions on the potential benefit of providing coverage for acupuncture therapy to treat low back pain, neck pain, or headache/migraine.

Our study has several limitations. First, data on severity and duration of reported pain were not available. Thus, we were not able to quantify the clinical effectiveness of treatments or the chronicity of the diagnosis of neck/back pain or headache/migraine. Additionally, pain severity may affect the selection of treatment (e.g., those with more severe pain may choose PT or other therapy). Second, our study may have underestimated healthcare utilization related to medication use and acupuncture services. Treatments not reported in claims, such as over-the-counter medications or acupuncture sessions paid out-of-pocket, could not be captured. These therapies not billed to the insurer may bias the results towards null. Third, we used ICD diagnosis codes to identify pain and comorbid conditions. Inaccurate or incorrect medical coding may underreport or overreport the prevalence of conditions. Fourth, we did not know the specific types of acupuncture used in each session as codes for these types were unavailable. There are a variety of techniques practiced and each may have had a different impact. Fifth, there has been a dramatic decrease in opioid prescribing over time [3] which would impact opioid initiation. However, this change should affect both acupuncture and comparison groups. Finally, we did not consider other therapies that were introduced during and after our study’s timeframe and could influence opioid prescribing.

Non-opioid treatments are increasingly encouraged in pain treatment practices to address risks of opioid addiction. This study aimed to address a gap in knowledge regarding acupuncture’s role in reducing opioid medications. We found a significant advantage for acupuncture in opioid use cessation compared to NSAIDs and PT overall. These results can help clinicians, patients, and decision-makers in treatment decisions and coverage. More research is needed on how acupuncture can be effectively integrated into therapeutic practice, especially on specific pain conditions, effects on emergency care, and comparisons to other types of non-opioid treatments. Specifically, randomized, controlled prospective trials are needed to establish whether observed changes in opioid medication or other resource use are the result of acupuncture treatment.

## Supplementary Data

Supplementary data are available at *Pain Medicine* online.

## Supplementary Material

pnab187_Supplementary_Data

## Notes

Funding source: The funding for this research was provided by Anthem, Inc.
