# Supplementary material for: Do Acupuncture Services Reduce Subsequent Utilization of Opioids and Surgical Interventions Compared to Noninvasive Therapies among Patients with Pain Conditions?
Source: Pain Med. 2021 Jun 15;22(11):2754–62. doi: 10.1093/pm/pnab187 (PMC8633741; doi:10.1093/pm/pnab187)
Supplement: pnab187_Supplementary_Data [file pnab187_supplementary_data.zip › Appendix Table 1.docx]

**Appendix Table 1. *Current Procedural Terminology (CPT)* or *Generic Product Identifier (GPI)* code for acupuncture and other therapies**

|  | CPT | GPI |
| --- | --- | --- |
| Acupuncture | 97810, 97811, 97813, 97814 |  |
| Physical therapy | 97010, 97012, 97016, 97018, 97022, 97024, 97026, 97028, 97033, 97034, 97035, 97036, 97039, 97110, 97112, 97113, 97116, 97124, 97127, 97139, 97140, 97150, 97161, 97162, 97163, 97164, 97165, 97166, 97167, 97168, 97169, 97170, 97171, 97172, 97530, 97533, 97535, 97537, 97542, 97545, 97546, 97597, 97598, 97602, 97605, 97606, 97607, 97608, 97610, 97750, 97755, 97760, 97761, 97763, 97799 |  |
| Nonsteroidal anti-inflammatory drugs |  | 6610x, 6699x, 6760x |
| Neck/back pain procedures: total disc arthroplasty, osteotomy, laminotomy, laminectomy, percutaneous vertebroplasty, arthrodesis, anterior/posterior instrumentation, pelvic fixation, spinal fusion, spinal fixation/reinsertion, insertion of biomechanical device(s), transpedicular decompression | 0095T, 0098T, 0163T, 0164T,  0165T, 0375T, 22856, 22857,  22858, 22861, 22862, 22864,  22865, 22210, 22206, 22207,  22208, 22212, 22214, 22216,  22220, 22222, 22224, 22226,  22532, 22533, 22534, 22548,  22551, 22552, 22554, 22556,  22558, 22585, 22590, 22595,  22600, 22610, 22612, 22614,  22614, 22630, 22632, 22632,  22633, 22634, 22800, 22802,  22804, 22808, 22810, 22812,  22830, 22840, 22841, 22849,  22853, 22842, 22843, 22844,  22845, 22846, 22847, 22848,  22853, 22854, 22859, 63001,  63003, 63005, 63012, 63015,  63016, 63017, 63045, 63046,  63047, 63048, 63050, 63051,  63180, 63182, 63185, 63190,  63191, 63194, 63196, 63198,  63200, 63250, 63252, 63265,  63267, 63270, 63272, 63275,  63277, 63280, 63282, 63285,  63287, 63290, 63020, 63035,  63040, 63043, 63030, 63035,  63042, 63044, 63055, 63056,  63057, 63075, 63076, 63081,  63082, 63085, 63086, 63300,  63304, 63308, 22510, 22511,  22512, 22513, 22514, 22515,  22818, 22819 |  |
| Headache/migraine procedures: rhytidectomy, septoplasty, ethmoidectomy, excision inferior turbinate, submucous resection, nasal/sinus endoscopy, occipital nerve block therapy (anesthetic injection), implantation/replacement of neurostimulator, percutaneous closure of interatrial communication, transection or avulsion of supraorbital/infraorbital nerve, and repair of brow ptosis | 15824, 15826, 30130, 30140, 30520, 31200, 31201, 31205, 31254, 31255, 64405, 64450, 64553, 64555, 64575, 64590, 64732, 64734, 67900, 93580 |  |
