# Supplementary material for: Do Acupuncture Services Reduce Subsequent Utilization of Opioids and Surgical Interventions Compared to Noninvasive Therapies among Patients with Pain Conditions?
Source: Pain Med. 2021 Jun 15;22(11):2754–62. doi: 10.1093/pm/pnab187 (PMC8633741; doi:10.1093/pm/pnab187)
Supplement: pnab187_Supplementary_Data [file pnab187_supplementary_data.zip › Appendix Table 2.docx]

**Appendix Table 2. Baseline demographics and clinical characteristics of patients using acupuncture and nonsteroidal antiinflammatory drugs/physical therapy (NSAIDs/PT) before propensity score matching**

|  | Acupuncture | NSAIDs/PT | Standardized difference |
| --- | --- | --- | --- |
| Sample size, n | 55,801 | 845,656 |  |
| Age, years, mean (SD) | 45.4 (13.11) | 46.8 (15.71) | 0.10 |
| Female, n (%) | 36,316 (65.1) | 467,485 (55.3) | 0.20 |
| Geographic region, n (%) |  |  | 1.33 |
| Northeast | 14,034 (25.2) | 110,747 (13.1) |  |
| West | 35,171 (63.0) | 192,736 (22.8) |  |
| Midwest | 1,614 (2.9) | 207,328 (24.5) |  |
| South | 3,210 (5.8) | 286,310 (33.9) |  |
| Missing/Unknown | 1,772 (3.2) | 48,535 (5.7) |  |
| Insurance plan type, n (%) |  |  | 0.31 |
| CDHP | 8,074 (14.5) | 175,901 (20.8) |  |
| HMO | 5,524 (9.9) | 144,629 (17.1) |  |
| PPO | 42,202 (75.6) | 525,086 (62.1) |  |
| Other | <10% | <10% |  |
| Urban/rural classification based on zip code, n (%) |  |  | 0.43 |
| Urban | 50,226 (90.0) | 626,605 (74.1) |  |
| Rural | 3,836 (6.9) | 170,597 (20.2) |  |
| Missing/Unknown | 1,739 (3.1) | 48,454 (5.7) |  |
| Proportions of Race/Ethnicity based on zip code, mean % (SD) |  |  | 0.61 |
| Asian | 13.3% (17.6%) | 4.7% (9.4%) |  |
| Black | 5.6% (12.1%) | 10.0% (18.2%) |  |
| White | 69.7% (23.9%) | 78.5% (22.5%) |  |
| Hispanic | 20.5% (21.7%) | 11.9% (18.1%) |  |
| Household income based on zip code, median (IQR) | $108,264 ($53,711) | $83,430 ($41,156) | 0.52 |
| Deyo-Charlson Comorbidity Index Score, mean (SD) | 0.3 (0.83) | 0.4 (0.95) | 0.08 |
| Deyo-Charlson Comorbidity Index Score Categories, n (%) |  |  | 0.13 |
| 0 | 43,254 (77.5) | 635,869 (75.2) |  |
| 1 | 8,771 (15.7) | 134,790 (15.9) |  |
| 2 | 2,338 (4.2) | 41,538 (4.9) |  |
| 3+ | 1,438 (2.6) | 33,459 (4.0) |  |
| **Comorbidities of interest, n (%)** |  |  |  |
| Depression | 7,066 (12.7) | 95,311 (11.3) | 0.04 |
| Anxiety | 9,147 (16.4) | 113,864 (13.5) | 0.08 |
| Substance use disorders | 767 (1.4) | 10,297 (1.2) | 0.01 |
| Other mental health | 6,606 (11.8) | 73,618 (8.7) | 0.10 |
| Number of pain diagnoses, mean (SD) | 2.4 (2.16) | 1.9 (1.86) | 0.25 |
| Relevant pain diagnoses |  |  |  |
| Back pain | 26,783 (48.0) | 382,054 (45.2) | 0.06 |
| Neck pain | 14,603 (26.2) | 161,104 (19.1) | 0.17 |
| Headache/migraine | 9,097 (16.3) | 132,420 (15.7) | 0.02 |
| Any opioid use, n (%) | 13,526 (24.2) | 212,417 (25.1) | 0.02 |
| Hospital admissions, mean (SD) | 0.1 (0.39) | 0.1 (0.42) | 0.04 |
| ED visits, mean (SD) | 0.2 (0.68) | 0.3 (0.78) | 0.08 |
| Number of unique medications (GPI-8), mean (SD) | 5.4 (5.41) | 4.9 (4.97) | 0.11 |
| CDHP = consumer-driven health plan; ED = emergency department; GPI-8 = Generic Product Indicator-8 digits; HMO = health maintenance organization; PPO = preferred provider organization; SD = standard deviation | | | |
