# Supplementary material for: Do Acupuncture Services Reduce Subsequent Utilization of Opioids and Surgical Interventions Compared to Noninvasive Therapies among Patients with Pain Conditions?
Source: Pain Med. 2021 Jun 15;22(11):2754–62. doi: 10.1093/pm/pnab187 (PMC8633741; doi:10.1093/pm/pnab187)
Supplement: pnab187_Supplementary_Data [file pnab187_supplementary_data.zip › Appendix Table 3.docx]

**Appendix Table 3. Effect of acupuncture on opioid use and dosage for physical therapy (PT) only subgroup analysis**

|  | **Acupuncture** | | | **PT** | | | **DID Absolute Difference** | **P value^a^** |
| --- | --- | --- | --- | --- | --- | --- | --- | --- |
|  | **Baseline Period** | **Follow-up period** | **Absolute difference** | **Baseline Period** | **Follow-up period** | **Absolute difference** |  |  |
| Naïve opioid users (without baseline opioid use) | | | | | | | | |
|  | **Acupuncture** | | | **PT** | | |  |  |
| Number of members, n (%) | 6,247 (15.9%) | | | 6,328 (16.0%) | | | -0.1 | 0.551 |
| Number of fills, mean (SD) | - | 1.59 (1.42) | - | - | 1.62 (1.41) | - | -0.03 | 0.047 |
| Total days' supplied, mean (SD) | - | 12.8 (25.4) | - | - | 13.4 (30.0) | - | -0.6 | 0.367 |
| Total quantity dispensed, mean (SD) | - | 61.8 (112.3) | - | - | 65.7 (127.5) | - | -3.9 | 0.016 |
| Total MME per day, mean (SD) | - | 37.9 (23.7) | - | - | 39.2 (24.7) | - | -1.3 | 0.003 |
| Above 50 MME per day threshold, n (%) | - | 1,503 (24.1%) | - | - | 1,640 (25.9%) | - | -1.8 | 0.016 |
| Continuous opioid users (with baseline opioid use) | | | | | | | | |
|  | **Acupuncture** | | | **PT** | | |  | **Adjusted *P* value^b^** |
| Number of members, n (%) | 5,878 (48.9%) | | | 5,960 (50.1%) | | | -1.2 | 0.064 |
| Number of fills, mean (SD) | 5.06 (5.67) | 5.08 (5.62) | 0.02 | 5.3 (6.1) | 5.5 (6.2) | 0.2 | -0.18 | 0.009 |
| Total days' supplied, mean (SD) | 107.6 (172.1) | 112.9 (176.1) | 5.3 | 122.4 (196.0) | 127.9 (195.4) | 5.5 | -0.2 | 0.809 |
| Total quantity dispensed, mean (SD) | 408.3 (688.6) | 417.8 (674.4) | 9.5 | 482.2 (865.1) | 499.0 (903.8) | 16.8 | -7.3 | 0.522 |
| Total MME per day, mean (SD) | 44.7 (75.9) | 46.4 (117.9) | 1.7 | 53.2 (113.9) | 52.9 (111.5) | -0.3 | 2.0 | 0.217 |
| Above 50 MME per day threshold, n (%) | 1,370 (23.3%) | 1,524 (25.9%) | 2.5 | 1,645 (27.6%) | 1,684 (28.3%) | 0.7 | 1.8 | 0.014 |
| ^a^P values generated from Wilcoxon-Mann-Whitney tests for continuous measures and chi-square goodness of fit tests for categorical measures  ^b^Adjusted *P* values for interaction term of therapeutic group and time were outputted from generalized estimating equation models  DID = difference in difference; MME = morphine milligram equivalents; NSAIDs = nonsteroidal anti-inflammatory drugs; PT = physical therapy; SD = standard deviation | | | | | | | | |
