# Supplementary material for: Do Acupuncture Services Reduce Subsequent Utilization of Opioids and Surgical Interventions Compared to Noninvasive Therapies among Patients with Pain Conditions?
Source: Pain Med. 2021 Jun 15;22(11):2754–62. doi: 10.1093/pm/pnab187 (PMC8633741; doi:10.1093/pm/pnab187)
Supplement: pnab187_Supplementary_Data [file pnab187_supplementary_data.zip › Appendix Table 4.docx]

**Appendix Table 4. Effect of acupuncture on healthcare utilization and total costs for physical therapy (PT) subgroup analysis**

|  | **Acupuncture (n = 51,428)** | | | **PT (n =51,428)** | | | **DID absolute difference** | **Adjusted *P* value^a^** |
| --- | --- | --- | --- | --- | --- | --- | --- | --- |
|  | **Baseline period** | **Follow-up period** | **Absolute difference** | **Baseline period** | **Follow-up period** | **Absolute difference** |  |  |
| All-cause |  |  |  |  |  |  |  |  |
| Inpatient hospitalization |  |  |  |  |  |  |  |  |
| N (%) | 3,254 (6.3) | 4,632 (9.0) | 2.7% | 3,287 (6.4) | 4,426 (8.6) | 2.2% | 0.5% | 0.06 |
| Count, mean (SD) | 0.1 (0.4) | 0.1 (0.5) | 0.03 | 0.1 (0.4) | 0.1 (0.5) | 0.03 | 0.0 | 0.40 |
| ED visits |  |  |  |  |  |  |  |  |
| N (%) | 7,470 (14.5) | 6,644 (12.9) | -1.6% | 7,314 (14.2) | 7,100 (13.8) | -0.4% | -1.2% | <0.001 |
| Count, mean (SD) | 0.2 (0.7) | 0.2 (0.7) | -0.02 | 0.2 (0.6) | 0.2 (0.7) | -0.0006 | -0.02 | <0.001 |
| Physician office visits |  |  |  |  |  |  |  |  |
| N (%) | 47,989 (93.3) | 49,982 (97.2) | 3.9% | 49,209 (95.7) | 49,588 (96.4) | 0.7% | 3.1% | <0.001 |
| Count, mean (SD) | 7.3 (7.5) | 9.7 (9.5) | 2.4 | 6.7 (6.6) | 7.6 (7.5) | 0.9 | 1.5 | <0.001 |
| Pain-related |  |  |  |  |  |  |  |  |
| Inpatient hospitalization |  |  |  |  |  |  |  |  |
| N (%) | 1,406 (2.7) | 1,802 (3.5) | 0.8% | 1,776 (3.5) | 1,965 (3.8) | 0.4% | 0.4% | 0.001 |
| Count, mean (SD) | 0.01 (0.1) | 0.03 (0.2) | 0.014 | 0.02 (0.2) | 0.03(0.2) | 0.010 | 0.004 | 0.006 |
| ED visits |  |  |  |  |  |  |  |  |
| N (%) | 3,976 (7.7) | 3,365 (6.5) | -1.2% | 3,977 (7.7) | 3,630 (7.1) | -0.7% | -0.5% | 0.011 |
| Count, mean (SD) | 0.1 (0.4) | 0.1 (0.4) | -0.02 | 0.1 (0.4) | 0.1 (0.4) | -0.01 | -0.01 | 0.001 |
| Physician office visits |  |  |  |  |  |  |  |  |
| N (%) | 33,949 (66.0) | 43,301 (84.2) | 18.2% | 35,922 (69.8) | 40,559 (78.9) | 9.0% | 9.2% | <0.001 |
| Count, mean (SD) | 2.7 (4.3) | 4.6 (6.6) | 1.9 | 2.2 (3.3) | 3.0 (4.0) | 0.8 | 1.1 | <0.001 |
| Total all-cause costs^b^ per patient, mean (SD) |  |  |  |  |  |  |  |  |
| Total medical and pharmacy costs | $9,765 ($31,650) | $13,422 ($31,397) | $3,657 | $9,590 ($30,335) | $11,569 ($33,969) | $1,979 | $1,678 | <0.001 |
| Medical costs | $8,609 ($30,689) | $11,828 ($30,423) | $3,219 | $7,937 ($28,712) | $9,778 ($32,040) | $1,841 | $1,378 | <0.001 |
| Hospitalization cost | $2,524 ($24,980) | $3,363 ($21,616) | $839 | $2,814 ($23,196) | $3,028 ($23,215) | $214 | $625 | 0.001 |
| ED cost | $577 ($2,553) | $513 ($2,411) | -$64 | $532 ($2,401) | $504 ($2,424) | -$28 | -$36 | 0.053 |
| Pharmacy costs | $1,855 ($7,535) | $2,136 ($10,070) | $281 | $2,005 ($10,186) | $2,176 ($10,476) | $171 | $110 | 0.02 |
| ^a^Adjusted *P* values for interaction term of therapeutic group and time were outputted from generalized estimating equation models  ^b^Members with third-party pharmacy coverage excluded  DID = difference in difference; ED = emergency department NSAIDs = nonsteroidal anti-inflammatory drugs; PT = physical therapy; SD = standard deviation | | | | | | | | |
